# In vitro antioxidant and antiproliferative effect of the extracts of *Ephedra chilensis* K Presl aerial parts

**DOI:** 10.1186/s12906-019-2462-3

**Published:** 2019-03-04

**Authors:** Marco Mellado, Mauricio Soto, Alejandro Madrid, Iván Montenegro, Carlos Jara-Gutiérrez, Joan Villena, Enrique Werner, Patricio Godoy, Luis F. Aguilar

**Affiliations:** 1 Instituto de Química, Facultad de Ciencias, Pontificia Universidad Católica de Valparaíso, Av. Universidad, #330 Valparaíso, Chile; 2 Departamento de Química, Universidad Técnica Federico Santa María, Valparaíso, Chile; 3 Departamento de Química, Facultad de Ciencias Naturales y Exactas, Universidad de Playa Ancha, Av. Leopoldo Carvallo, #270 Valparaíso, Chile; 4 Escuela de Obstetricia y Puericultura, Facultad de Medicina, Universidad de Valparaíso, Ángamos #665, Reñaca, Viña del Mar, Chile; 5 Centro de Investigaciones Biomédicas (CIB), Escuela de Medicina, Universidad de Valparaíso, Viña del Mar, Chile; 6 Centro de Investigaciones Biomédicas (CIB), Laboratorio de Investigación-Estrés Oxidativo, Facultad de Medicina, Universidad de Valparaíso, Valparaíso, Chile; 7 Departamento De Ciencias Básicas, Campus Fernando May Universidad del Biobío, Chillán, Chile; 8 Instituto de Microbiología Clínica, Facultad de Medicina, Universidad Austral de Chile, Valdivia, Chile

**Keywords:** *Ephedra chilensis*, Antioxidant activity, Cytotoxic effect, Selectivity, MCF-7 and PC-3

## Abstract

**Background:**

*Ephedra chilensis* K Presl, known locally as pingo–pingo, is a Chilean endemic plant used in traditional medicine as an anti-inflammatory and used in other treatments. However, unlike for the other *Ephedra* species, there have been no reports on the antioxidant and cytotoxic effects of this plant. The present study aims to explore the potential applications of *E. chilensis* extract as a cytotoxic agent against in vitro cancer cell lines and to explore the relationship between this extract and antioxidant activity.

**Methods:**

Total anthraquinone, flavonoid, and phenolic contents, as well as antioxidant activity (DPPH, FRAP, and TRAP assays) and cytotoxic effect on several cancer cell lines (MCF-7, PC-3, DU-145, and HT-29) were measured for the hexane, dichloromethane and ethanol extracts of *E. chilensis*. In addition, several correlations among the phytochemical content, antioxidant activity, and cytotoxic effect were evaluated. Finally, GC-MS analyses of the most active extracts were carried out to identify their major components and to relate these components to the cytotoxic effect.

**Results:**

Antioxidant activity was found in the EtOH extracts of *Ephedra*, and the results were correlated with the phenolic content. For the cytotoxic activity, the non-polar extracts of *E. chilensis* had the highest antiproliferative effect for the MCF-7 and PC-3 cancer lines; the extract was shown to be up to three times more selective than doxorubicin. However, the cytotoxic effect was not correlated with the antioxidant activity. Lastly, the GC-MS analysis showed a high concentration of saturated fatty acids (mainly *n*-hexadecanoic acid) and terpenoids (mainly 4-(hydroxy-ethyl)-γ-butanolactone).

**Conclusion:**

The cytotoxic activity and selectivity of the non-polar extracts of *E. chilensis* for the MCF-7 and PC-3 cell lines could be related to the terpenic compounds and fatty acids of the extracts or to the synergistic effect of all of the compounds in the extracts. These non-polar extracts can be used for the development of new drugs against breast and prostate cancer.

## Background

There has been an ongoing increase in the incidence of chronic non-communicable diseases (CNCDs) worldwide, with cancer as one example of such diseases [1]. In fact, the World Health Organization estimated that in the year 2030, 11 million people will die due to cancer [2]. The traditional chemotherapy treatment against cancer causes undesirable side effects, and complementary and alternative medicine (CAM) has thus emerged as a possible solution. Among CAM treatments, phytotherapy is currently the most commonly used [3]. Several studies have focused on the natural antioxidant intake because oxidation is closely related to cancer development [4].

The genus *Ephedra* is the only genus in the Ephedraceae family (which contains 35 to 45 species in total, commonly found worldwide) [5]. This genus has been studied due its high contents of ephedrine alkaloids [6]. However, several secondary metabolites as alkaloids (amphetamine-type, imidazole, quinoline, pyrrolidine, and others), flavonoids (flavonols, dihydroflavonol, flavanone, flavanols, flavones, anthocyanin), tannins (dimmer, trimmer and tetramer of proanthocyanidins), lignans, naphthalenes, esters, terpenoids, phenolic acids, and quinones have been reported in the Ephedra genus plants [7]. In addition, some *Ephedra* species have anti-inflammatory, antiviral, hepatoprotective, antibacterial and antifungal activities, as well as anticancer activities [7]. In fact, *E. foeminea* and *E. alata* are used in CAM for cancer treatment in south-eastern Europe [8]. By contrast, Chile has one such species, namely *Ephedra chilensis* K Presl, commonly known as pingo-pingo [9]. It is particularly abundant in the central zone and has a pink fruit that is fleshy and edible [10]. The ethnopharmacological information showed that *E. chilensis* is used for treating ulcers, abscesses, and clearing pus, as an astringent, anti-inflammatory, diuretic, and tonic, and for treatment of colds, and stomach and bladder pain, and is beneficial in the treatment of asthma, gonorrhoea, and syphilis [10]. Additionally, potential bioactive applications of *E. chilensis* have been explored. Examples of such applications are sun protection properties and growth-inhibitory activity against some bacterial cultures [11, 12].

Despite the previous ethnopharmacological applications of *E. chilensis*, no studies have been reported on its effect on cancer or on the antioxidant capacity of this species. Therefore, the goals of this work are to measure the phytochemical content (anthraquinones, phenols, and flavonoids), to evaluate the antioxidant activity and the cytotoxicity against cancer cells (MCF-7, HT-29, PC-3, and DU-145) and non-tumour (CoN) lines for different *E. chilensis* extracts, and to identify the chemical composition (GC-MS analysis) of the extracts that present the greatest activity against the cancer cell lines.

## Methods

### Plant material

The plant was collected at the coordinates 33° 05′ 50″ S – 71° 35′ 27″ W at 460 m.a.s.l. in April 2016. A voucher specimen is kept in the Herbarium of Natural Products Laboratory of Universidad de Playa Ancha, Valparaíso, Chile (ECKP-2016). The plant was recognized by Rodrigo Villaseñor, Biology professor and expert in botany. He considered the plant’s morphological properties.

### Extraction procedure

The portion of the plant selected (aerial parts) was dried at room temperature and then subjected to successive extractions using different solvents of increasing polarity, similar to a procedure reported in a previous study [13]. Using dried plant (310 g) and 1 L of each solvent (n-hexane (Hex), dichloromethane (CH_2_Cl_2_) and ethanol (EtOH)), the extraction of *E. chilensis* was completed in 48 h, and replicated three times. All of the obtained extracts were concentrated in a rotary evaporator at 40 °C, and then each extract was stored at room temperature in the dark.

### Phytochemical determination

#### Total anthraquinones content estimation

This estimation was carried out using the protocol of Arvouet-Grand et al. adapted by Mellado et al [14]. One mL of 2% *w*/*v* aluminium trichloride (AlCl_3_) in ethanol was mixed with the same volume of the extract solution in ethanol (1.0 mg/mL). The mix was incubated for 10 min at room temperature, and absorbance was measured at 486 nm against a blank sample consisting of 1.0 mL extract solution with 1.0 mL of methanol without AlCl_3_. The absorbance values were interpolated using an emodin calibration curve (0–70 mg / L). The total anthraquinones content was expressed as μg of emodin equivalents (EE) / g of dry extract. All of the determinations were performed in triplicate.

#### Total flavonoid content estimation

The total flavonoid content was determined using the Dowd method, as adapted by Arvouet-Grand et al. [15]. One mL of 2% *w*/*v* aluminium trichloride (AlCl_3_) in ethanol was mixed with the same volume of the extract solution in ethanol (1.0 mg/mL). The mix was incubated for 10 min at room temperature, and absorbance was measured at 415 nm against a blank sample consisting of a 1.0 mL extract solution with 1.0 mL of methanol without AlCl_3_. The absorbance values were interpolated using a quercetin calibrate curve (0–100 mg / L). The total flavonoid content was expressed as μg of quercetin equivalents (QE) / per g of dry extract. All of the measurements were replicated three times.

#### Total phenolic content determination

The amount of total phenolic compounds in the extracts was determined using the method reported by Waterman et al. with small modifications determined by our research team [13]. Each extract sample (2.0 mg) was diluted to 2.0 mL with ethanol. Five hundred microliters were mixed with a Folin-Ciocalteau reagent (2.5 mL, 0.2 N) and incubated for 5 min. Then, a 7.5% *w*/*v* Na_2_CO_3_ solution (2.0 mL) was added and the mix was incubated in the dark at room temperature for 2 h. The absorbance of the solution was measured at 700 nm using ethanol as the blank. The obtained absorbance values were interpolated using a Gallic acid standard curve (0–200 mg / L) and the total phenolic content was expressed as mg of Gallic acid equivalents (GAE) per g of dried extract. All of the measurements were replicated three times.

### Antioxidant capacity

#### Radical scavenging assays using DPPH

The DPPH assay was performed as described by Brand-Williams et al, with modifications [16]. The sample (100 μL, extracts at 0–10 mg / mL) was mixed with a 50 μM DPPH^●^ solution (2.9 mL) freshly prepared in ethanol. A 50 μM DPPH^●^ solution (2.9 mL) with ethanol (0.1 mL) was used as the control. The sample and control solutions were incubated for 15 min at room temperature, and the absorbance was measured at 517 nm. The inhibition (%) was calculated by the following equation:

1 $$ \mathrm{Inhibition}\ \left(\%\right)=100\%\times \left({\mathrm{A}}_{\mathrm{control}}-{\mathrm{A}}_{\mathrm{sample}}\right)/{\mathrm{A}}_{\mathrm{control}}\Big) $$

From the obtained Inhibition (%) values, the IC_50_ value was determined by linear regression analysis.

#### Ferric reducing antioxidant power (FRAP) assay (III)

The ferric reducing power was measured as described by Dudonné et al. with modifications [17]. Freshly prepared (10 volumes of 300 mM acetate buffer, pH 3.6, with 1.0 volume of 10 mM TPTZ (2,4,6-tri(2-pyridyl)-s-triazine) in 40 mM hydrochloric acid, and 1.0 volume of 20 mM ferric chloride) FRAP reagent (3.0 mL) was mixed with deionized water (300 μL) and the sample (100 μL, 1.0 mg/mL of each extract). The mix was incubated for 30 min at 37 °C in a water bath and the absorbance was measured at 593 nm using ethanol as the blank solution. The obtained absorbance values were interpolated in a Trolox calibrate curve (0–200 mg / L) and the FRAP values were expressed in mM Trolox equivalent antioxidant capacity (mM TEAC). All of the measurements were performed in triplicate.

#### Total reactive antioxidant power (TRAP) assay

The method developed by Romay et al. was slightly modified for this experiment [18]. One volume of 10 mM solution of ABAP (2,2′-azo-bis(2-amidino propane) was mixed with the same volume of 150 μM solution of ABTS (2,2′-azinobi(3-ethylbenzothiazoline-6-sulphonic acid) using PBS 100 mM at pH of 7.4 (TRAP solution). The mixture was incubated at 45 °C for 30 min and then cooled to room temperature for use. Sample solution (10 μL, 1.0 mg / mL of each extract) was mixed with the TRAP solution (990 μL), and the absorbance was measured after 50 s at 734 nm against the ABTS solution as the blank. The absorbance values were interpolated in a Trolox standard curve (0–120 mg / L). All of the measurements were replicated three times.

#### Cell lines

The following experimental cell cultures were obtained from the American Type Culture Collection (Rockville, MD, USA): MCF-7 (human breast cancer), HT-29 (human colon cancer), PC-3 and DU-145 (human prostate cancer) and CoN (human colon epithelial cells CCD 841). All of the cell lines were grown in a DMEM-F12 medium containing 10% FCS, 100 U/mL penicillin, 100 μg/mL streptomycin and 1 mM glutamine. The cells were seeded into 96 well microliter plates at 100 μL, with a plating density of 3 × 10^3^ cells/well. After a 24 h incubation at 37 °C (under a 5% humidified carbon dioxide ambient to allow cell attachment), the cells were treated with different concentrations of drugs and incubated for 72 h under the same conditions. A stock solution of extracts was prepared in ethanol, and the final concentration of this solvent was kept constant at 0.1%. Control cultures received only 0.1% ethanol.

#### In vitro growth inhibition assay

Following the method of Skehan et al., the sulforhodamine B assay was used with modifications [19, 20]. Briefly, the cells were seeded at 3 × 10^3^ cells per well of a 96-well, flat-bottomed 200 μL microplate. The cells were incubated at 37 °C in a 5% humidified CO_2_ ambient plus 95% air mixture and were treated with the extracts at different concentrations for 72 h. At the end of the drug exposure, the cells were fixed with 50% trichloroacetic acid at 4 °C (TCA final concentration 10%). After washing with distilled water, the cells were stained with 0.1% sulphorhodamine B (Sigma-Aldrich, St. Louis, MO, USA), dissolved in 1% acetic acid (50 μL/well) for 30 min, and then washed with 1% acetic acid to remove the unbound stain. The protein-bound stain was solubilized with a 10 mM unbuffered Tris base (100 μL). The cell density was determined using a fluorescence plate reader (wavelength 540 nm). Untreated cells were used as the negative control while cells treated with doxorubicin (Doxo.) were used as the positive control. In addition, all of the samples were tested from 0 to 10 μg/mL (concentration of extracts) using ethanol as the carrier solvent. All of the measurements were replicated three times. Finally, Sigma Plot software was used to calculate the IC_50_ value.

#### Selectivity index

The selectivity of each extract in each cell line was analysed by calculating the selectivity index (SI) as IC_50_ CoN / IC_50_ cancer cell line. If the values of SI were equal or greater than 3, it is said that the extract is selective. If the value exceeds 10, the selectivity was assumed to be very high [21].

#### GC-MS analysis

All extracts of *E. chilensis* were analysed by GC-MS (Shimadzu GC-17 A, mass detector GC-MS-QP5050, Shimadzu Corp, Kioto, Japan). The extracts (1.0 μL) were injected in the splitless mode (5 min) into a BPX-5MS column (30 m, 0.25 mm diameter, SGE) with helium as the carrier gas at a constant flow of 1.5 mL min^− 1^ and a column pressure of 92.3 KPa. The Injector temperature was 260 °C. The thermal profile was as follows: the temperature was held for 2 min at 80 °C and then increased at a rate of 8 °C /min^− 1^ to 270 °C, and then that temperature was maintained for 15 min. The mass scan was set between 35 and 500 a.m.u. The mass spectra were compared with the internal spectra database. A match below 90% confidence was considered as “unknown compounds”. Compounds in the chromatograms were identified by comparison of their mass spectra with those in the NIST/EPA/NIH Mass spectral Library [22]. Chromatographic peaks were considered “unknown” when their similarity index (MATCH) and reverse similarity index (RMATCH) were less than 850 and were discarded [23]. These parameters refer to the matching capability of the target spectrum with the standard spectrum in the NIST Library (a value of 1000 indicates a perfect fit). Additionally, a comparison of the retention index was made with values reported in the literature for the same type of column, or with commercial standards when available [24]. The retention indexes were determined under the same operating conditions in relation to a homologous *n*-alkanes series (C_8_–C_36_) by the equation:

2 $$ \mathrm{RI}=100\times \left(\left(\mathrm{n}+\mathrm{T}{\mathrm{r}}_{\left(\mathrm{unknown}\right)}-\mathrm{T}{\mathrm{r}}_{\left(\mathrm{n}\right)}\right)/\left(\mathrm{T}{\mathrm{r}}_{\left(\mathrm{N}\right)}-\mathrm{T}{\mathrm{r}}_{\left(\mathrm{n}\right)}\right)\right) $$

where n is the number of carbon atoms in the smaller *n*-alkane, N is the number of carbon atoms in the larger *n*-alkane, and Tr was the retention time. The relative concentrations of the components were obtained by peak area normalization.

#### Statistical analysis

The data were reported as the mean values ± standard deviation (SD). Due to non-parametric data, a Kruskal-Wallis ANOVA was used with a confidence level of 95% with STATISTICA 7.0 program.

## Results

### Phytochemical content

After *E. chilensis* extracts were obtained, their phytochemical content (total anthraquinone, flavonoid, and phenolic contents) was measured using colorimetric assays as summarized in Table 1. For the total anthraquinones and flavonoids content, there are significant differences in the CH_2_Cl_2_ extracts (*p* < 0.05) compared to the other extracts. The total phenolic content in both CH_2_Cl_2_ extract and EtOH extract shows significant differences (*p* < 0.05) with the Hex extract.

**Table 1:** Phytochemical contents of different extracts of the aerial part of *E. chilensis*

Extract	Percentage yield of the extract (%)	Anth (μg EE / g d.e.)	Flav (μg QuE / g d.e.)	Phen (mg GAE / g d.e.)
Hex	0.19	4.42 ± 0.00^ab^	23.58 ± 0.01^ab^	7.06 ± 0.03^a^
CH_2_Cl_2_	0.51	15.65 ± 0.17^a^	95.14 ± 0.04^a^	31.16 ± 1.62^ab^
EtOH	3.16	2.30 ± 0.04^b^	5.92 ± 0.02^b^	42.18 ± 1.24^b^

Different letters in the same column indicate significant differences; *p* < 0.05, *n* = 3

### Antioxidant activity

The antioxidant activity of *E. chilensis* extracts was evaluated in a series of in vitro tests using the DPPH, FRAP and TRAP assays (see Table 2). The DPPH assay showed that the Hex extract had poor activity (*p* < 0.05) compared with the positive controls (Trolox and Gallic acid). CH_2_Cl_2_ and EtOH extracts show similar activities, and these activities are different from the activities of Trolox and Gallic acid (*p* < 0.05). For the FRAP assay, the CH_2_Cl_2_ and EtOH extracts show better antioxidant activity than the positive controls (*p* < 0.05). Finally, the TRAP assay showed that the Hex extract was the least active of all of the tested extracts compared with the positive controls (Gallic acid and BHT) with significant differences (*p* < 0.05).

**Table 2:** Antioxidant activity of the aerial part of *E. chilensis* extracts and positive controls

Extract / sample	DPPH (IC_50_ mg / mL)	FRAP (TEAC mM)	TRAP (TEAC mM)
Hex	13.77 ± 0.37^a^	3.90 ± 0.20^a^	0.28 ± 0.05^a^
CH_2_Cl_2_	3.02 ± 0.02^b^	21.05 ± 0.18^b^	1.40 ± 0.07^b^
EtOH	0.68 ± 0.01^c^	24.00 ± 0.43^b^	1.53 ± 0.06^b^
Trolox	0.11 ± 0.01^d^	n.a.	n.a.
Gallicacid	0.06 ± 0.01^d^	1.72 ± 0.02^c^	1.13 ± 0.01^b^
BHT	n.a.	1.52 ± 0.07^c^	1.06 ± 0.02^b^

Different letters in the same column indicate significant differences; *p* < 0.05, *n* = 3; *n.a* Not applicable

### Phytochemical content - antioxidant activity relationship

The correlation between the phytochemical content and antioxidant activity was evaluated using Pearson’s Correlation Coefficient (r). All of the obtained correlations are summarized in Table 3. In this case, all of the antioxidant assays were closely related to the total phenolic content (r > 0.9 and *p* < 0.05), while anthraquinones and flavonoids were not related to this property (r < 0.9 and *p* > 0.05).

**Table 3:** Pearson’s correlation coefficient (r) for phytochemicals and antioxidant activity

Phytoconstituent	DPPH	FRAP	TRAP
Anthraquinones	− 0.056	0.097	0.119
Flavonoids	−0.082	0.118	0.147
Phenols	−0.942*	0.928*	0.934*

*Significant differences (*p* < 0.05)

### Cytotoxic activity

The cytotoxic activity of *E. chilensis* extracts was evaluated using a colorimetric assay, in vitro against different cancer cell lines, namely MCF-7 breast cancer, HT-29 colon cancer, DU-145 and PC-3 prostate cancer, and one non-tumour cell line of human colon epithelial cells CCD 841 (CoN). IC_50_ values were obtained from this assay and are summarized in Table 4. For the MCF-7 and PC-3 cancer cell lines, Hex and CH_2_Cl_2_ showed more activity than Doxorubicin (*p* < 0.05), while for the HT-29 and DU-145 cell lines, all of extracts were less active than Doxorubicin (*p* < 0.05). Finally, for the non-cancer cell line (CoN), CH_2_Cl_2_ had the most active extract.

**Table 4:** Cytotoxic effect of *E. chilensis* extracts (IC_50_ μg / mL) for different cell lines compared to an antineoplastic drug

Extract/Sample	MCF-7	HT-29	PC-3	DU-145	CoN
Hex	0.31 ± 0.02^a^	3.67 ± 0.18^a^	0.43 ± 0.11^a^	4.25 ± 0.51^a^	2.82 ± 0.85^a^
CH_2_Cl_2_	0.28 ± 0.09^a^	2.07 ± 0.39^a^	0.38 ± 0.18^a^	1.93 ± 0.58^b^	1.19 ± 0.66^a^
EtOH	1.29 ± 0.13^b^	*i*	5.02 ± 2.45^b^	4.96 ± 0.12^a^	5.05 ± 0.60^b^
Doxo.	1.10 ± 5.0.12^b^	0.55 ± 0.05^b^	2.75 ± 0.50^c^	0.55 ± 0.06^c^	5.50 ± 0.53^b^

Different letters in the same column indicate significant differences; *p* < 0.05, *n* = 3; *i* = inactive at maximal concentration 10 μg / mL; Doxo. = Doxorubicin

### Phytochemical content - antioxidant activity – cytotoxic effect relationships

The correlations between the cytotoxic effects on the MCF-7 and PC-3 cancer cell lines, phytochemical content, and antioxidant activity were evaluated out using Pearson’s Correlation Coefficient (r). The results are shown in Table 5. It was found that in this assay, the phytochemical content and antioxidant activity are not correlated with the cytotoxic effect on both cancer cell lines (r < 0.9 and *p* > 0.05).

**Table 5:** Pearson’s Correlation Coefficient (r) between the Phytochemicals and the Antioxidant Activity with MCF-7 and PC-3cancer cell lines

Cancer cell line	Phytochemical content			Antioxidant activity		
	Ant	Phen	Flav	DPPH	FRAP	TRAP
MCF-7	−0.299	0.135	−0.430	− 0.2847	0.289	0.247
PC-3	−0.352	0.262	−0.455	−0.356	− 0.363	0.333

### Selectivity of cytotoxic effect

The selectivity measurement was carried out using a ratio between the non-cancer cell line and the cancer cell line (IC_50_CoN / IC_50_ cancer cell line). The results of the selectivity for *E. chilensis* extracts are presented in Table 6. For the MCF-7 and PC-3 cancer cell lines, the *n*-hexane extract showed more selectivity than Doxorubicin (1.8 times in MCF-7 and 3.3 times in PC-3). However, no extracts were selective against HT-29 and DU-145 (selectivity ≤1.0).

**Table 6:** Selectivity index of extracts and positive control of theMCF-7, HT-29, PC-3, and DU-145 cancer cell lines

Extract/Sample	MCF-7	HT-29	PC-3	DU-145
Hex	9.1	0.8	6.6	0.6
CH_2_Cl_2_	4.3	0.6	3.1	0.6
EtOH	3.9	n.c.	1.0	1.0
Doxorubicin	5.0	10.0	2.0	10.0

*n.c* Not calculated

### GC-MS analysis

The most active and selective *E. chilensis* extracts against breast and prostate cancer cell lines (Hex and CH_2_Cl_2_) were analysed by GC-MS. The results of this analysis are shown in Tables 7 and 8. In the Hex extract, high amounts of fatty acids and derivatives were found. Among these, we found *n-*tetradecanoic, *n*-pentadecanoic, *n*-hexadecanoic, and *n*-octadecanoic acid (44.37% of the total extract composition, see Table 7). In the CH_2_Cl_2_ extract, we found *n*-tetradecanoic and *n*-hexadecanoic acid ethyl ester with 2.4% of the total composition (see Table 8). Alcohol derivatives of fatty acids were found in both extracts (*trans*-9-hexadecen-1-ol and 1-Eicosanol). For the ethanol extract, compounds with match quality higher than 65% could not be identified.

**Table 7:**
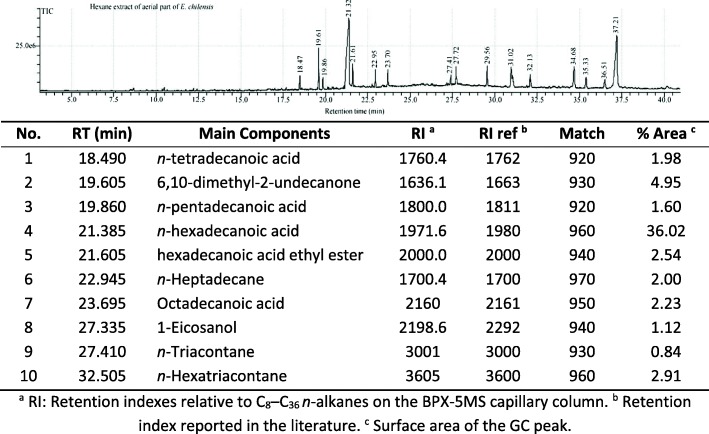
GC-MS analysis for the *n*-hexane extract

**Table 8:**
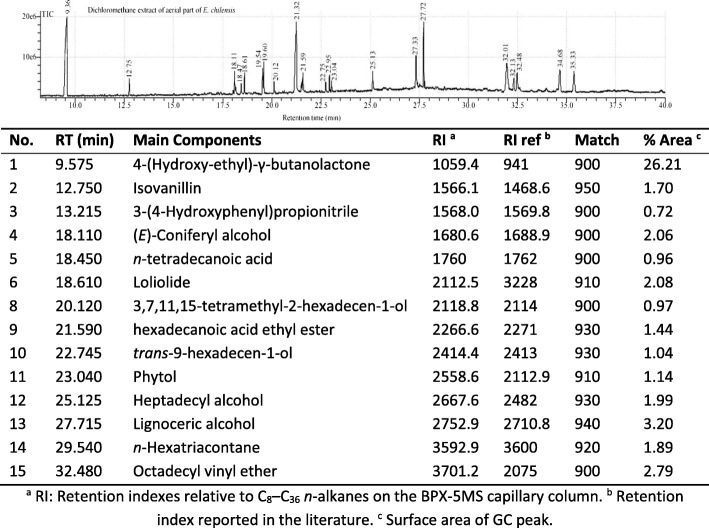
GC-MS analysis for the CH_2_Cl_2_ extract

In addition, terpenic compounds were identified in the CH_2_Cl_2_ extract. Among these, we found phytol, loliolide, and 4-(Hydroxy-ethyl)-γ-butanolactone (29.43% of the total extract composition, see Table 8). Furthermore, the long-chain alkanes family was another compound family identified in GC-MS analysis. Among these compounds, we found *n*-heptadecane, *n*-triacontane and *n*-hexatriacontane (see Tables 7 and 8) with 5.75% of the Hex extract composition and 1.89% of the CH_2_Cl_2_ extract composition.

Other families of compounds identified in GC-MS were phenolic compounds that were found only in the CH_2_Cl_2_ extract, namely isovanillin and (*E*)- Coniferyl alcohol with 3.76% of the extract composition (see Table 8).

## Discussion

The anthraquinone and flavonoid contents are concentrated in the CH_2_Cl_2_ extracts (*p* < 0.05, see Table 1), and comparing these results with other *Ephedra* species, we found that there are no reports of anthraquinones in any *Ephedra* species. However, flavonoids and related compounds have been reported in *E. aphylla*, *E. sinica*, *E. campylopoda*, and *E. alata* [7]. Furthermore, the obtained flavonoid content showed a three-fold decrease compared to that of *E. major* [25]. In addition, phenolic compounds were mainly found in the CH_2_Cl_2_ and EtOH extracts (see Table 1), and our results for both of these extracts have similar values to those of *E. major*, [25] and a higher phenolic content than *E. sinica* [26]. These compound types have not been reported in *E. chilensis* (except ephedrine). However, other *Ephedra* species have been isolated and some of these compounds have been identified [25, 27].

The antioxidant activity of *E. chilensis* extracts was evaluated in a series of in vitro tests (see Table 2). The DPPH assay showed that EtOH extracts of *E. chilensis* have the highest antioxidant activity, and present better activity than *E. laristanica* and *E. Sarcocarpa* (IC_50_ = 4.6 and IC_50_ = 5.3 mg / mL, respectively) [28, 29]. Despite these results, all extracts have lower antioxidant activity than the positive controls. The FRAP and TRAP assays showed that the Hex extract has less antioxidant activity than the CH_2_Cl_2_ and the EtOH extracts. However, FRAP assay showed that all extracts are more active than the positive control (between 2.1 and 17.3 times more active than Gallic acid). TRAP assay showed that the extracts have similar activity to the positive controls. In addition, phytochemical content has been associated with the antioxidant activity [30, 31]. The DPPH scavenging activity found in the present work is consistent with the total phenolic content (r = − 0.942, see Table 3), which is similar to the previous reports on *E. sinica* [32]. For the FRAP and TRAP assays, we found correlations between the total phenolic content (r > 0.9, *p* < 0.05, see Table 3). Based on the results obtained in the FRAP assay, the *E. chilensis* extracts manifest their antioxidant effect as reductive substances acting as single electron transferrers [33]. The TRAP assay on *E. chilensis* extracts showed great affinity of the extract with the peroxyl radical [34].

The cytotoxic effect of *E. chilensis* extracts was evaluated in vitro against several cell lines (see Table 4). The *E. chilensis* extracts present higher activity for the MCF-7 and PC-3 cell lines (IC_50_ < 1.0 μg / mL), while the HT-29 and DU-145 cell lines are resistant to the same treatment (IC_50_ > Doxo in each cell line, see Table 4). The cytotoxic effect is related to several factors among which we can mention the solvent used for the extraction and the species; e.g.*,* decoctions of *E. foeminea* and *E. alata* are used for breast cancer treatment in south-eastern Europe [8]. *E. alata* extract has no cytotoxic effect against human liver cancer or against the leukaemia cell line, [35] *E. sinica* alcoholic extracts has a low cytotoxic effect against melanoma and non-cancer, [36] and the CHCl_3_ extract of *E. viridis* does show cytotoxic action against leukaemia cells [37]. The values obtained for MCF-7 and PC-3 cancer cell lines show promising anticancer properties for possible drug discovery and development according to the American National Center Institute [21]. In fact, the non-polar extracts possess more activity than the antineoplastic drug for the MCF-7 and PC-3 cancer cell lines (as much as 3.9 times higher activity for MCF-7 and up to 7.2 times higher for PC-3, see Table 4). Exogenous antioxidant intake could be associated with chemoprevention of chronic diseases such as cancer, together with the presented correlation between phytochemical content and the antioxidant activity [31, 38]. The correlation between the phytochemical content of *E. chilensis* extracts and the cytotoxic effect was evaluated, but it was found that there was no relationship between the antiproliferative effect and the phytochemical content or antioxidant activity (r < 0.5, see Table 5). In addition to the cytotoxic effect, the selectivity for the cancer cell lines as a possible drug development pathway is a very relevant feature that must be evaluated considering the undesirable side effects of traditional chemotherapy [39]. In this regard, the Hex and CH_2_Cl_2_ extracts of *E. chilensis* have better selectivity index values than doxorubicin (up to 1.8 times more selective for MCF-7 and 3.3 times more selective for PC-3, see Table 6).

Based on the above discussion, the Hex and CH_2_Cl_2_ extracts were analysed by GC-MS (see Table 7 and Table 8). The analysis did not identify ephedrine, which is a typical compound of this species. The ephedrine concentration in *Ephedra* species is variable; e.g.*, E. major*, *E. fragilis*, *E. distachya,* and *E. monosperma* have different ephedrine concentrations, while this alkaloid was not identified in *E. tweediana* and *E. foeminica* [40, 41]. Nevertheless, the effect of ephedrine as a cytotoxic compound is not important because comparing both extracts (those free of ephedrine and those that do have ephedrine), similar activity is observed for non-small cell lung cancer [42]. For breast cancer cells, ephedrine has a poor cytotoxic activity [43]. Moreover, the GC-MS of both *E. chilensis* non-polar extracts (Hex and CH_2_Cl_2_) showed high concentrations of long-chain fatty acids, and some of these have been identified in other *Ephedra* species, e.g.*, n*-tetradecanoic, *n*-pentadecanoic, *n*-hexadecanoic, and *n*-octadecanoic acids were found in seeds of *E. nevadensis*, *E. viridis*, *E. przewalskii*, *E. geradiana*, *E. campylopoda*, and *E. sinica*, and in the leaves of *E. equizetina* [44–46]. Regarding the cytotoxic effect, these fatty acids can inhibit abnormal breast cancer cells [47]. In fact, *n*-tetradecanoic acid, *n*-dodecanoic acid, and *n*-octadecanoic acid have differentiation and/or cytotoxic and/or apoptotic effects on breast cancer cells [48–50]. *n*-octadecanoic acid has cytotoxic effects for prostate carcinoma [51, 52]. For *n*-hexadecanoic acid, there have been no reports of cytotoxic activity on breast or prostate cancer cells. However, *n*-hexadecanoic acid affects colon cancer cell growth, while its ethyl ester derivative can inhibit the DNA topoisomerase I and is an apoptosis inductor in leukaemia and neuroblastoma cells [53–55]. Furthermore, alcohol derivatives of fatty acids such as *trans*-9-hexadecen-1-olare only present in the CH_2_Cl_2_ *E. chilensis* extract. This compound has a growth inhibition effect on breast cancer [56]. Other fatty alcohol derivatives such as 1-Eicosanol and lignoceric alcohol have not been reported to show cytotoxic effects on breast or prostate cancer. Nevertheless, they show antiproliferative activity for other cancer cell lines [57, 58]. Regarding the fatty acids and their derivative content in non-polar *Ephedra* extract, they could be related with activity and selectivity due to the *n*-tetra, *n*-penta, *n*-hexa, and *n-*octadecanoic acids present in the hexane extract corresponding to 41.37% of the total extract (see Table 7). The dichloromethane extract has fewer *n*-tetra and *n*-octadecanoic acids (2.40% of total extract, see Table 8). However, the synergistic effect between the fatty acids and other secondary metabolites cannot be ruled out.

Terpenoid derivatives were also identified, mainly in the dichloromethane extract of *E. chilensis* (see Table 8). Among these, we found phytol which was also found in *E. campylopoda* [59]. This compound showed cytotoxic activity for a wide range of cancer cell lines, and particularly for the MCF-7 and PC-3 cancer cell lines [60]. Others terpenic derivatives such as 4-(hydroxy-ethyl)-γ-butanolactone and loliolide were identified in the same extract (CH_2_Cl_2_, see Table 8). These compounds have highly similar structures. The extracts that present loliolide have high activity for the breast cancer MCF-7 cell line [61]. Moreover, in the same extract, phenolic compounds such as isovanillin and (*E*)-coniferyl alcohol were identified (see Table 8). Similar compounds (vanillin, ferulic acid, and lignin) have been found in *E. breana* and *E. alata* [62, 63]. These compounds have a common core (benzene-3,4-OR) which is a fragment present in several molecules with antiproliferative activity such as lignins and benzaldehydes [64–66].

On the other hand, in both non-polar extracts, we identified long-chain alkanes such as *n*-heptadecane, *n*-triacontane, and *n*-hexatriacontane (see Table 7 and Table 8), of which *n*-heptadecane and *n*-triacontane had cytotoxic effects on several cancer cell lines, with a particularly pronounced effect on breast cancer observed for *n*-triacontane [67, 68].

Other compounds present in the Hex extract such as 6,10-dimethyl-2-undecanone and 3-(4-hydroxyphenyl)propionitrile were identified (see Table 7). The first compound has a cytotoxic effect on the breast cancer cell lines [69]. A compound similar to 3-(4-hydroxyphenyl)propionitrile presents β-oestrogen receptor-selective inhibition which is important for breast cancer cells [70, 71].

Finally, despite the above discussion of the cytotoxic effect of the principal components identified in the non-polar extracts, the synergic effect between them is not ruled out.

## Conclusions

In conclusion, hexane and dichloromethane extracts of *E. chilensis* showed low antioxidant activity but high activity for MCF-7 and PC-3 cancer cell lines. However, there was no correlation between the antioxidant activity and the anticancer activity. In both extracts, we found significant amounts of fatty acids and derivatives, and terpenic and phenolic compounds were identified by the GC-MS technique. All of the compounds presented a cytotoxic effect on many cancer cell lines, mainly breast and prostate cancer lines. Nevertheless, the synergic effect between these compounds is not ruled out.

These promising results suggest that non-polar *E. chilensis* extracts could be a source for new drug discoveries against breast and prostate cancers. These new drugs could have significantly milder secondary effects compared to chemotherapy.

## Abbreviations

Ant: Anthraquinones content
CAM: Complementary and alternative medicine
CH_2_Cl_2_: Dichloromethane extract
CNCDs: Chronic non-communicable diseases
CoN: Human colon normal cell line
Doxo: Doxorubicin drug
DPPH: 2,2-Diphenyl-1-picrylhydrazyl free radical assay
DU-145: Human prostate carcinoma cell line
EtOH: Ethanol extract
Flav: Flavonoid content
FRAP: Ferric Reducing Antioxidant Power assay
GC-MS: Gas chromatography – Mass Spectrometry coupled assay
Hex: n-Hexane extract
HT-29: Human colorectal adenocarcinoma cell line
IC_50_: Half Inhibition concentration
MCF-7: Human breast adenocarcinoma cell line
PC-3: Human prostate adenocarcinoma cell line
Phen: Phenolic content
TRAP: Total Reactive Antioxidant Power assay
